# Version by the Vertex in Shoulder Presentations

**Published:** 1877-05-20

**Authors:** William H. Bolling

**Affiliations:** Professor of Midwifery in Hospital College of Medicine Louisville, Ky.


### VERSION BY THE VERTEX IN SHOULDER PRESENTATIONS.

By WILLIAM H. BOLLING, M. D., Professor of Midwifery in Hospital College of Medicine Louisville, Ky.

About twenty years ago Dr. M. B. Wright, of Cincinnati, published an article advising version by the vertex in shoulder presentations by means of a method now known as Braxton Hicks', after a London physician who subsequently described the same operation, viz: "Previous to the rupture of the membranes, with one hand within the vagina to thrust up the shoulder, the external hand to push the head from the iliac region to center of superior strait, and then to allow nature to terminate the labor."

Some eight or nine years ago I was called to the assistance of a midwife, and found a poor woman suffering from protracted labor, the right hand of the now dead child protruding from the vulva, (the bones of the right arm were broken in many places by the efforts of the midwife to force a delivery,) the shoulder and portion of the chest external to the womb; the child's back was toward mother's abdomen, its head lying across the center of the left ilio-pectineal line; the uterus was acting violently, and the mother was much exhausted. I brought her well under the influence of chloroform, and by its relaxing effect was enabled without effort to return the protruded parts within the uterus; my external hand through the flaccid abdominal and uterine walls readily recognized the head, and forced it down to take the place of the receding shoulder; in a few moments uterine contraction completed the labor naturally. The wom n made a good recovery.

On the night of March 9th, I was again summoned to the assistance of a midwife, and found a woman exhausted from a prolonged labor. The left hand of the almost lifeless child protruded from the vulva; the shoulder and portion of the chest tightly grasped by the os uteri, which from its irritable condition seemed in a state of almost continuous contraction; the back of child was toward the mother's abdomen, the head across the center of right ilio-pectineal line. I brought the woman profoundly under the influence of chloroform, and *without violence* returned the extruded parts, and moved the shoulder sufficiently high to allow me with my external hand to press the occiput from its abnormal position to a point opposite the right acetabulum, my internal hand securing the proper flexion. The labor then progressed as if the case had originally been one of the second position of the vertex. The child, although with difficulty resuscitated at birth, is now doing well, as is also its mother.

My object in citing the above cases is to call attention to the feasibility of version by the vertex even under the most unfavorable conditions. My success in this limited number of such advanced cases prompts me to believe that the time has arrived when version by the feet, at least in shoulder presentations, should be regarded as an unwarranted operation, as it certainly is a dangerous one to both mother and child. *Previous to the rupture of the membranes* rectifying a transverse presentation is comparatively a simple matter when we follow Dr. Wright's plan, and in addition gain the assistance of gravity by placing the woman in the knee-and-chest position, as is done in prolapsus of the cord.

